# Quantum Oscillations in Ferromagnetic (Sb, V)_2_Te_3_ Topological Insulator Thin Films

**DOI:** 10.1002/adma.202102107

**Published:** 2021-08-31

**Authors:** Liguo Zhang, Toni Helm, Haicheng Lin, Fengren Fan, Congcong Le, Yan Sun, Anastasios Markou, Claudia Felser

**Affiliations:** ^1^ Max‐Planck Institute for Chemical Physics of Solids Nöthnitzer Str. 40 01187 Dresden Germany; ^2^ Dresden High Magnetic Field Laboratory (HLD) Helmholtz‐Zentrum Dresden–Rossendorf (HZDR) Bautzner Landstr. 400 01328 Dresden Germany

**Keywords:** ferromagnetic order, Landau fan diagram, magnetically doped topological insulator, molecular beam epitaxy, quantum oscillation

## Abstract

An effective way of manipulating 2D surface states in magnetic topological insulators may open a new route for quantum technologies based on the quantum anomalous Hall effect. The doping‐dependent evolution of the electronic band structure in the topological insulator Sb_2−_ *_x_* V*_x_* Te_3_ (0 ≤ *x* ≤ 0.102) thin films is studied by means of electrical transport. Sb_2−_ *_x_* V*_x_* Te_3_ thin films were prepared by molecular beam epitaxy, and Shubnikov–de Hass (SdH) oscillations are observed in both the longitudinal and transverse transport channels. Doping with the 3d element, vanadium, induces long‐range ferromagnetic order with enhanced SdH oscillation amplitudes. The doping effect is systematically studied in various films depending on thickness and bottom gate voltage. The angle‐dependence of the SdH oscillations reveals their 2D nature, linking them to topological surface states as their origin. Furthermore, it is shown that vanadium doping can efficiently modify the band structure. The tunability by doping and the coexistence of the surface states with ferromagnetism render Sb_2−_ *_x_* V*_x_* Te_3_ thin films a promising platform for energy band engineering. In this way, topological quantum states may be manipulated to crossover from quantum Hall effect to quantum anomalous Hall effect, which opens an alternative route for the design of quantum electronics and spintronics.

## Graphical abstract

A systematic study of electric transport in Sb_2−_ *_x_* V*_x_* Te_3_ films demonstrates the coexistence of topological surface states and long‐range ferromagnetism evidenced by Shubnikov–de Haas oscillations. Vanadium doping acts as an efficient tuning parameter for the Fermi surface size. This opens up a new route for the investigation of the crossover between different topological states based on this material class.

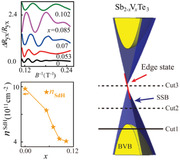

## 1 Introduction

Topological insulators (TIs) have been investigated extensively over the past decade owning to their nontrivial surface states.^[^ ^1^, ^2^, ^3^, ^4^, ^5^ ^]^ As they are topologically protected, these states bear a huge potential for high‐speed electronics as well as spintronics applications.^[^ ^6^, ^7^, ^8^, ^9^ ^]^ Ferromagnetic (FM) order can be introduced via 3d‐element dopants. Hence, the broken time‐reversal symmetry may enable the detection of the quantum anomalous Hall effect (QAHE) due to the presence of dissipationless edge states.^[^ ^10^, ^11^, ^12^ ^]^ The combination of such devices with conventional superconductors were predicted to host Majorana fermions, which are suitable for braiding devices for use in topological quantum computers.^[^ ^13^, ^14^ ^]^ As the band structure of real materials is complex, it is challenging to realize the QAHE or Majorana fermions at a higher temperature. Highly precise band structure engineering is required to suppress the contribution of the bulk bands effectively. To date, this has posed one of the main limiting barriers to the development of practical devices based on the QAHE. Therefore, a deeper understanding of how the band structure of TI can be artificially designed is inevitable.

Shubnikov–de Hass (SdH) oscillations are a quantum coherence phenomenon commonly observed in clean metals, where the charge carriers can complete at least one full cyclotron motion without impurity scattering under magnetic field.^[^ ^15^ ^]^ Wealth parameters such as the Fermi surface topology and mean‐free path can be extracted from the oscillation period and the temperature‐dependent amplitude variation.^[^ ^16^ ^]^ Quantum oscillations have been extensively used as a tool to study high temperature superconductors and topological materials.^[^ ^17^, ^18^, ^19^, ^20^ ^]^ The recent observation of the three‐dimensional (3D) quantum Hall effect (QHE) in ZrTe_5_ has attracted further enthusiasm to study of quantum oscillations in TI materials.^[^ ^21^ ^]^ Quantum oscillations have been observed in binary compounds, Bi_2_Se_3_, Bi_2_Te_3_, and Sb_2_Te_3_ bulk crystals and thin flakes.^[^ ^22^, ^23^, ^24^, ^25^ ^]^ In these systems, the oscillations originate from either the surface states or the bulk bands, depending on the position of the chemical potential.^[^ ^26^ ^]^ Recently, quantum oscillations were discovered in 3d elements doped TI single crystals, such as Fe‐doped Sb_2_Te_3_ and V doped (Bi, Sn, Sb)_2_(Te, S)_3_.^[^ ^24^, ^27^ ^]^ However, no long‐range FM order was observed. The results motivated the preparation of thin films of similar materials with the potential for FM order coexisting with high mobility topological surface states.

So far, to our knowledge, there are only a few reports on the observation of quantum oscillations in magnetically doped TIs, such as V‐doped (Bi, Sb)_2_Te_3_, Sm‐doped Bi_2_Se_3_^.[^ ^28^, ^29^ ^]^ However, in all of those studies, the oscillations were only observed in longitudinal resistance. Furthermore, the systematical studies on the mechanism of the quantum oscillations in magnetically doped TIs is still lacking. In this work, we prepared V‐doped Sb_2_Te_3_, in which quantum oscillations were observed for both *R_xx_* and *R_yx_* component, coexisting with long range FM order for the first time with the sample dimension in millimeter scale. Such quantum oscillation proves the improved quality of the samples. More importantly, the stronger quantum oscillations in Hall channel comparing to the *R*_xx_ channel makes it a platform to perform the investigation of different quantized Hall effect tuned by gating voltage or chemical doping. Our studies shed light on the research of robust topological edge transport, topological magnetoelectric effect, and spintronic device applications based on this material.

## 2 Results and Discussion

**Figure 1:**
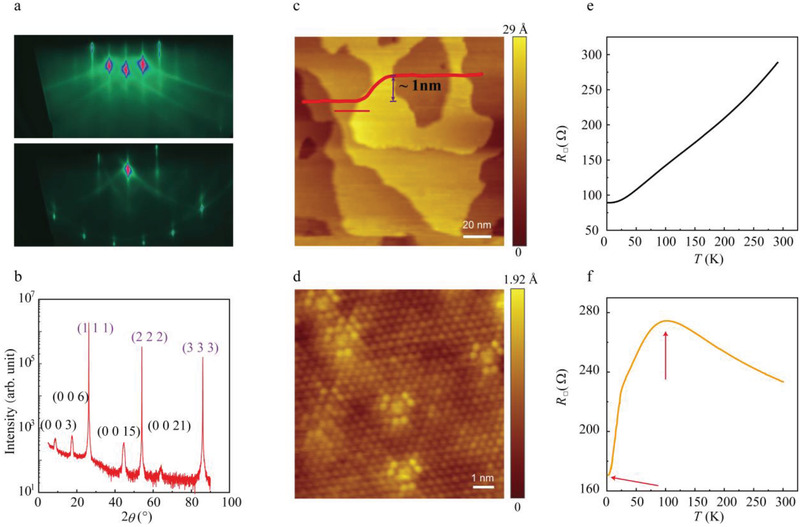
a) RHEED patterns along high symmetry direction, Γ‐M and Γ‐K of Sb_1.93_V_0.07_Te_3_. b) X‐ray diffraction data for (a). c) STM image of the topography (500 mV, 20 pA, *T* = 300 K), the inset shows the profile scan indicated by the red line in the figure. d) Atomic resolution image (500 mV, 100 pA, *T* = 300 K) of the *x* = 0.07 film. e,f) *RT* data of the pure and *x* = 0.07 Sb_2−_ *_x_* V*_x_* Te_3_ films.

In this work, two different substrates, InP (111) and STO (111), were selected to prepare the thin films using molecular beam epitaxy (mentioned in Supporting Information). **Figure** **1**a–d display the characterizations of Sb_2−_ *_x_* V*_x_* Te_3_ film with thickness, *t* = 44 nm and *x* = 0.07, grown on the InP (111)B surface. The reflection high‐energy electron diffraction (RHEED) patterns obtained from two high‐symmetry directions (Figure 1a) demonstrate that the film is well oriented along the in‐plane direction, and the high order patterns further confirm the high quality of the film. No additional signatures of other phases, but Sb_2−_ *_x_* V*_x_* Te_3_ and the substrate appear in the X‐ray diffractogram (Figure 1b). The micro area of the film was characterized with a room temperature scanning tunneling microscope (STM). The flat terrace structure with a step height of approximately 1 nm (Figure 1c) corresponds to the quintuple‐layer spacing along the *c* axis. The special shape of the defects is discernible in the atomic resolved image of the flat terrace, indicating that the main defect type is V substituting Sb, that is V_Sb_, as illustrated in Figure 1d.^[^ ^30^ ^]^

**Figure 2:**
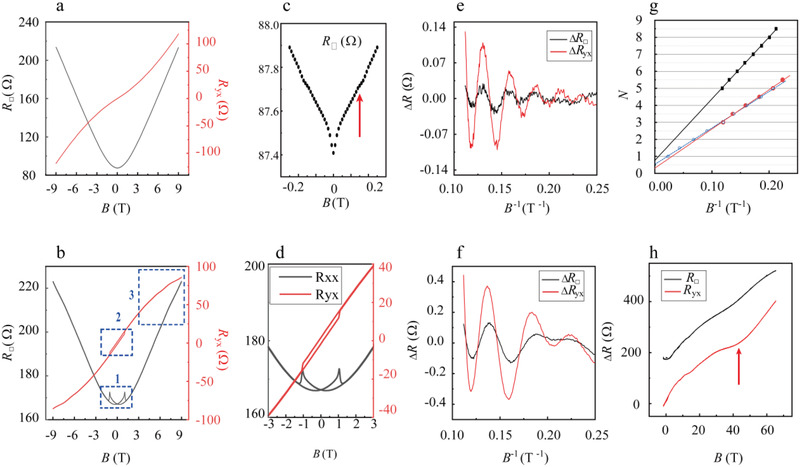
The sheet and Hall resistances as a function of magnetic field of a) the pure and b) *x* = 0.07 Sb_2−_ *_x_* V*_x_* Te_3_ film, with *t* = 44 nm, at 2 K. The dashed rectangles 1, 2, 3 represent the behavior of *R*_◽_ in the low‐field range, the anomalous Hall effect in the low‐field range, and SdH quantum oscillations in the high‐field range, respectively. c) the WAL MR behavior in low‐field region in pure sample; d) the hysteresis loops of the doped sample in MR and Hall resistance confirm the FM order in the doped sample. e,f) SdH oscillations up to 9 T at 2 K for both longitudinal and Hall resistance channels after background subtraction from (a) and (b), respectively. g) Landau fan diagram with maxima and minima positions taken from (e) and (f) marked by black solid squares and red solid circles, respectively. The blue open circles are extracted from the data of the doped sample shown in (f). h) The sheet and Hall resistances of the *x* = 0.07 film measured in pulsed field of up to 65 T, at 0.6 K. The arrow indicates the quantum limit.

To investigate the effects of the dopants, electrical transport measurements are performed on the respective samples. In the pure Sb_2_Te_3_ film with a thickness of 44 nm, the resistance (*R*) versus temperature (*T*) curve, *RT* curve, presents a typical metallic behavior with a residual‐resistance ratio (*R* (300 K)/*R* (2 K)) of approximately 3 (Figure 1e). This value matches previous reports on high‐quality Sb_2_Te_3_.^[^ ^25^ ^]^ With the introduction of slight V dopants (*x* = 0.07), the *RT* curve exhibits a strikingly different overall shape compared to the undoped film with the same thickness. The *RT* curve exhibits three distinct regions (Figure 1f): In the high‐temperature region, *R* shows insulating behavior until a maximum at approximately 107 K. This insulating behavior is possibly caused by the V‐3d impurity state above the Fermi level (see more analyses in Supporting Information).^[^ ^31^, ^32^ ^]^ Crossing the transition point, *R* drops rapidly until it starts to slow down again around 3 K. This behavior is preserved for a wide doping concentration and thickness range, as illustrated in Figure S2, Supporting Information. The extremely different temperature dependence for the undoped and slightly V‐doped Sb_2_Te_3_ films indicates a strong tailoring effect of the V dopants on the band structure of Sb_2_Te_3_. This is further supported by the following magneto‐transport measurements. **Figure** **2**a depicts the field dependence of the longitudinal and transverse resistance of the undoped Sb_2_Te_3_ film (44 nm). The sheet magnetoresistance (*R*_◽_) of the film exhibits positive magnetoresistance (MR) in the whole magnetic field range. And in low‐magnetic field (below 0.2 T), a weak anti‐localization (WAL) behavior was shown in Figure 2c, which originates from the nontrivial Berry phase that is present in the materials.^[^ ^33^ ^]^ Simultaneously, the Hall resistance shows a nonlinear behavior due to multiband contributions, as reported in a previous study.^[^ ^25^ ^]^ Interestingly, in the V‐doped film, Sb_1.93_V_0. 07_Te_3_ (44 nm), additional features are manifested (Figure 2b). In the low‐field region (−2 T < *B* < 2 T), the *R*_◽_ curve has a “butterfly” shape (region 1 marked by the dashed rectangle), owning to a hysteresis between the up and down field sweep. Its origin lies in the nucleation process of the magnetic domains during the magnetization process in a FM material. At higher field, the positive MR behavior is recovered. As expected, the Hall resistance, *R*_yx_, exhibits an anomalous Hall component at low field (region 2). Figure 2d clearly shows the hysteresis behavior correspond to the FM order. Accompanied by nonlinear behavior upon increasing the field, clear quantum oscillations are discernible in the high‐field region (region 3). For both samples, in‐high magnetic field, they show a nearly linear MR, which is possible due to the multicarrier effect or the guiding center diffusion mechanism.^[^ ^34^, ^35^ ^]^

A low‐order polynomial is fitted to the slowly varying background and subtracted off each curve for the analyses of the oscillating component. The background‐subtracted data are shown in Figure 2e,f (further details are provided in Figure S4, Supporting Information). For both the undoped and doped sample, clear SdH oscillations are observed, presented in Figure 2e,f. In previous oscillation studies on pure Sb_2_Te_3_ films, theses oscillations were only observed in the *R*_xx_ channel.^[^ ^25^ ^]^ The observation of strong oscillations in both transport channels for both of the investigated samples proves the high quality of the films studied in the work at hand. Moreover, the oscillation amplitudes for the V‐doped film are even larger than those for the pure one. The extracted positions of the oscillation maxima and minima indicated in Figure 2e,f exhibit a similar periodicity in 1/*B*, that is, the same frequency (*F*) that corresponds to the size of the underlying cyclotron orbit, respectively. In these two samples, the oscillations of the two channels are almost in phase, which is most likely a coincidence (two examples with phase difference are shown in Supporting Information). In the following analyses, the Hall channel is selected, as its oscillation amplitude is stronger. The *F* is approximately 36.5 T and 23.2 T at 2 K for the pure and doped sample, respectively. The oscillation frequency is directly linked to the Fermi surface size.^[^ ^15^ ^]^ This implies a significant tailoring effect on the band structure induced by V doping, which is consistent with the conclusion from the *RT* curve. To gain a deeper understanding of the dopant influences, the positions of the extrema are plotted as a function of the Landau level index in Figure 2g (further details can be found in Figure S4, Supporting Information).^[^ ^25^, ^36^, ^37^ ^]^ According to previous studies, the positions that correspond to a minimum in the oscillating component are selected as the integer *N*, while the maximum positions are the half integer. For the pure sample, the first oscillation is resolved starting from *N* = 8.5, while the doped sample starts from *N* = 5.5 above 4 T field. It should be noted that as the oscillation amplitude is weaker in the longitudinal transport channel, all data shown in Figure 2g were only extracted from the Hall channel. The Landau level index is higher in the pure case (black data). The oscillation phase was extracted from the intercept of the linear fitting curve at 1/*B* = 0 in the Landau fan diagram. It is 0.74 (0.30) for the pure (doped) sample. For an ideal topological insulator, the predicted oscillation phase should be 0.5 due to the nontrivial band structure. According to recent studies, higher harmonics are required to distinguish topological Fermi surface from nontopological one.^[^ ^38^, ^39^ ^]^ In our case, nonlinear band dispersions, band gaps, and contributions from other bulk bands, can complicate the interpretation.^[^ ^40^ ^]^ This paper will focus on the frequency of the oscillations, which reflects the size of the Fermi surface directly.

For the same doped film, measurements were performed in a pulse magnet providing a magnetic field of up to 65 T at a temperature of 0.6 K, see Figure 2h. SdH oscillations are resolved in both transport channels. Especially, in the Hall resistance curve, a wide plateau forms around 43 T, which corresponds to the *N* = 1 Landau‐level index. This indicates that the quantum limit is reached. The Landau level indices were also extracted from the pulsed‐field data (shown in Figure S4, Supporting Information), as indicated by the blue open circles in Figure 2g. For the doped sample, *F* = 22 T, which is only slightly smaller as compared to the value at 2 K, and also the phase has changed compared to the 2 K data. The effective carrier density was deduced from the Onsager relation:^[^ ^15^ ^]^

(1) $$\begin{array}{c} F=\frac{2\pi \hbar n^{\text{SdH}}}{e} \end{array}$$

where *e* is the elementary charge, ℏ is the reduced Planck constant and *n* ^SdH^ is the effective carrier density. The *n* ^SdH^ values of the undoped and doped sample determined at 2 K are 9.28 × 10^11^ and 5.62 × 10^11^ cm^−2^, respectively. In comparison, the effective carrier densities, *n* ^Hall^, extracted from the slope of the low‐field Hall resistance (up to 2 T), are 7.01 × 10^13^ and 4.81 × 10^13^ cm^−2^, respectively, which are larger than the corresponding *n* ^SdH^. The difference may account for the additional transport channels causing unquantized plateaus and nonlinear Hall effect owning to extra bands, as discussed later in the paper.

**Figure 3:**
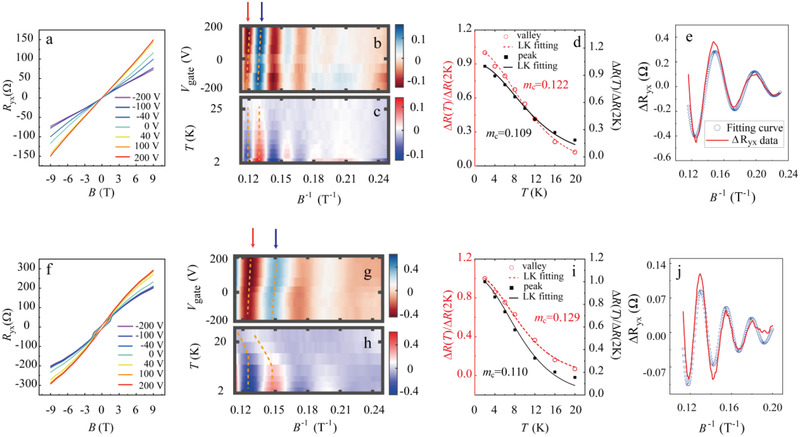
a,f) Hall resistances as a function of magnetic field tuned by bottom gate voltage of pure and *x* = 0.07 films with *t* = 33 and 22 nm, respectively. b,c,g,h) Color maps of the oscillating component of the Hall resistance of sample (a) and (f). b,g) depending on *V*_G_ recorded at *T* = 2 K and c,h) on temperature recorded for *V*_gate_ = 0 V. d,i) LK effective‐mass plots for the pure and *x* = 0.07 films at the oscillation maximum (black) and minimum (red) indicated in (c) and (h), respectively. e,j) Dingle fitting of oscillations for undoped and doped samples at 2 K.

In order to tune the chemical potential, films were deposited on SrTiO_3_ (111) substrates, which serves as the dielectric separation layer for the bottom gate.^[^ ^41^ ^]^ In this manner, an external gate voltage could actively control the charge carrier density. **Figure** **3**a,f presents the Hall data of the Sb_2_Te_3_ (*t* = 33 nm) and Sb_1.93_V_0.07_Te (*t* = 22 nm) films, recorded at 2 K for values of the bottom gate voltages, *V*_G_. When increasing *V*_G_, the carrier density becomes more dilute in the *p*‐type material, and hence the ordinary Hall resistance increases. The nominal *n* ^Hall^ is plotted against *V*_G_ in Figure S6, Supporting Information. With a gating bias from −200 V to 200 V, the carrier density *n* ^Hall^ of undoped (doped) film can be reduced from 6.65 × 10^13^ cm^−2^ (2.33 × 10^13^ cm^−2^) to 3.82 × 10^13^ cm^−2^ (1.61 × 10^13^ cm^−2^). For improved visibility of the gating effect, the background‐subtracted SdH oscillations are displayed in a color map in Figure 3b,g. The quantity of Δ*R*_yx_ (*B*)/*R*_yx_ (9 T), is used to represent the quantum oscillation amplitude. The following discussion will focus on the *T* and *V*_G_ dependence of Δ*R*_yx_ (*B*)/*R*_yx_ between 6 and 9 T, the field range where features are most pronounced and thus best distinguishable in the recorded data set. Two features are observed for both samples: First, the oscillation maximum and minimum at the highest field (indicated by the arrows) are strongest near 200 V. Second, the positions of these extrema exhibit a continuous shift toward a higher value of *B* ^−1^ with an increasing *V*_G_ (orange dashed lines). This behavior is consistent with the Onsager relationship. However, *F* changes only weakly with gate tuning (see Figure S6, Supporting Information).

Furthermore, the oscillations subside as the temperature is increased, (see Figure 3c,h). However, there is a clear difference between the two investigated samples. In the case of the undoped sample the positions of the extrema remain almost constant over the full temperature range, but they shift toward a higher field for the doped sample. This shift is accompanied by a reduction of the period, that is, a growth in *F*, which indicates a dramatic temperature‐dependent change of the band structure in the doped sample, thereby confirming the findings of a recent report.^[^ ^42^ ^]^ Nevertheless, the origin thereof remains unclear. One possibility is that a small gap separates the Fermi level and the V‐3d impurity states. The thermal activation effect can change of the size of the Fermi surface. The other scenario is that for the V‐doped sample, temperature can have a significant effect on the magnetization and the band structure. Hence, with increasing temperature, the film tends toward the pure sample that exhibits higher‐frequency oscillations. *V*_G_ is a powerful tool for tuning *n* ^Hall^ and the SdH‐oscillation amplitude. However, it only has a weak effect on *F*. A plausible explanation could be that the carriers that contribute to the SdH effect possess only a small effective mass, indicating the cross‐section of the Fermi surface changes slowly with the shift of the chemical potential. With increasing the temperature, the amplitude of the oscillations decreases sharply. The effective cyclotron mass of the charge carriers of the films can be determined via the Lifshitz–Kosevich (LK) formula:^[^ ^17^, ^25^ ^]^

(2) $$\begin{array}{c} \Delta R\propto R_{T}R_{D}\cos\left(2\pi F/B-\gamma \right) \end{array}$$

where *R*_T_ = *αT*/*B* sinh(*αm*_c_ *T*/*B*) and *R*_D_ = exp(−*αm*_c_ *T*_D_/*B*) are the temperature and scattering damping factors, respectively, α = 14.69 T K^−1^, *m*_c_ is the effective cyclotron mass in units of the free electron mass, *m*_e_, *T*_D_ is the Dingle temperature and γ is the temperature‐ and field‐independent Onsager phase. The maximum and minimum at the highest field (marked by dashed lines) in Figure 3c,h are selected for the cyclotron mass plots displayed in Figure 3d,i. For the pure film, the extracted values for the maximum and minimum are *m*_c_ = 0.109 ± 0.001 and *m*_c_ = 0.122 ± 0.002, in units of *m*_e_, respectively. These values are consistent with a previous report.^[^ ^25^ ^]^ For the doped sample, the same analysis is applied ignoring the shift of the position of the extrema. The extracted mass values, *m*_c_ = 0.110 ± 0.004 and *m*_c_ = 0.129 ± 0.003 are almost the same as in the pure case. Furthermore, the Dingle damping with field was analyzed. The respective fits are presented in Figure 3e,j. From the fitting, *T*_D_ is approximate to (20 ± 1) K and (16 ± 1) K, respectively, for the two samples. The scattering time, τ = ℏ/2*πk*_B_ *T*_D_ is, approximately 60 fs (72 fs). The larger scattering time enhances the oscillation amplitude.

**Figure 4:**
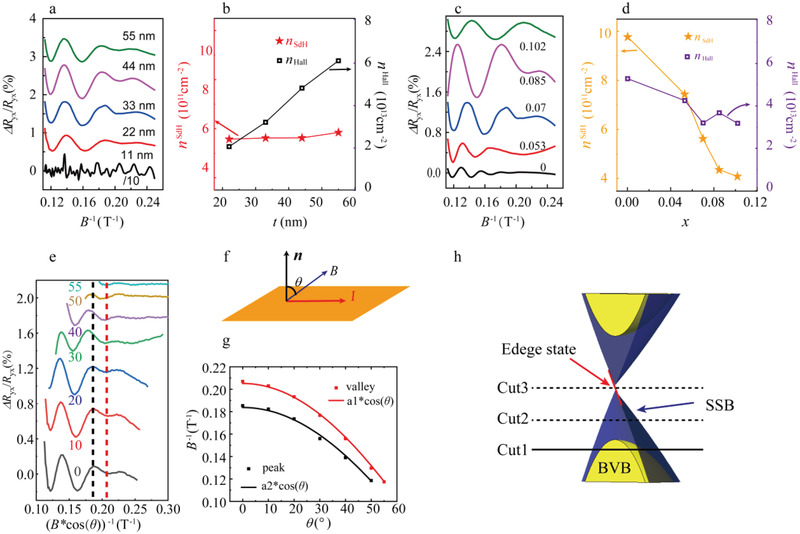
a) SdH oscillations in the Hall resistance channel of *x* = 0.07 films with various thickness *t*. b) Evolution of the charge carrier densities, *n* ^SdH^ and *n* ^Hall^ with varying thickness *t*. c) SdH oscillations in the Hall resistance channel of Sb_2−_ *_x_* V*_x_* Te_3_ films with *t* = 33 nm for varying *x*. d) Charge carrier densities, *n* ^SdH^ and *n* ^Hall^ plotted against *x*. e) SdH oscillation evolution depending on θ. f) Illustration of the transport measurement configuration with respect to the field orientation. g) cos(*θ)* fits to the positions of the extrema indicated in (e). h) Schematic band structure of the V‐doped Sb_2−_ *_x_* V*_x_* Te_3_ with different topological phases obtained by tuning of the chemical potential. Horizontal lines, “Cut1,” “Cut2,” and “Cut3,” indicate three different positions of the Fermi level achievable by band engineering.

Motivated by the different Landau level indices for the doped and undoped samples further *V* concentrations and film thicknesses were investigated. The quantum oscillation data for *x* = 0.07 and varying values of the thickness *t* are shown in **Figure** **4**a. (The oscillation curves are shifted for clarity). It should be noted that the oscillations disappear in the film with *t* ≤ 11 nm, which is likely caused by surface‐scattering effects.^[^ ^43^ ^]^ Furthermore, *n* ^Hall^ increases with thickness, while the *n* ^SdH^ stays almost constant (see Figure 4b). This shows that the cross section of the bands at the Fermi level contributed to the oscillations experiences little change with varying thickness. A detailed *x*‐dependence study was carried out in films with a fixed thickness of *t* = 33 nm. Apparently, *x* has a strong effect on the oscillation frequency—*F* reduces as *x* is increased (see Figure 4c). Nevertheless, while *n* ^SdH^ decreases rapidly, *n* ^Hall^ is only weakly affected (see Figure 4d). One thing should be noted that, in Figure 4c, the oscillation of period of the sample with *x* = 0.102 is not constant in 1/*B*. The reason is that significant Zeeman spin‐splitting has a strong effect on the band structure in this magnetic film with largest magnetization.

The key distinction between two‐dimensional (2D) and 3D Fermi surfaces can be observed in the angle‐dependence of quantum oscillations.^[^ ^15^ ^]^ Therefore, a film with *t* = 22 nm was investigated depending on the field orientation. A series of Hall measurements were conducted at various fixed tilt angles, θ, that is the angle between the external magnetic field and the substrate normal (illustrated in Figure 4f). In Figure 4e, the background‐subtracted oscillations are plotted against the inverse out‐of‐plane field component (B*cos(*θ)*)^−1^_._ The positions of the extrema (vertical dashed lines) remain constant in this plot, evidencing that the oscillation frequency scales neatly with cos(θ). This is further highlighted by the fits in Figure 4g. The angle dependence supports the assumption that the oscillations in the doped samples originate from the 2D topological surface bands. According to the conclusion above, the surface states remain unchanged with varying *t*. In contrast, V doping could “pull down” the surface state with respect to the Fermi level. With an increasing *x*, the cross section of the surface state becomes smaller, although there is still a heavy contribution from the bulk bands to the transport. This also explains the enhancement of the oscillation of the doped films compared to the undoped one. In the doped samples, the Fermi level is located closer to the Dirac point (there is a small gap in our case). The cyclotron radius, *r*_c_ ≈ μ/e*v*_F_ *B*, where μ is the chemical potential with respect to the Dirac point and *v*_F_ is the Fermi velocity, is much smaller in the doped samples.^[^ ^35^ ^]^ In this way, the oscillation signal is enhanced in the doped samples due to the reduced scattering events. A brief cartoon is given in Figure 4h to illustrate the band structure of the doped films. The horizontal line at “Cut1” represents the Fermi level observed in the current study. Here, the Fermi surface is comprised of bulk valence bands (BVB) and the 2D surface‐state band (SSB). This state is evidenced by the observed magnetic quantum oscillations. It can be expected that with a stronger band‐structure modulation, the Fermi level is tuned toward the state indicated by “Cut2” and ultimately “Cut 3”. For the “Cut2” case, only the SSB touches the Fermi level, consequently QHE should arise in an external magnetic field. Recently, the QHE has been achieved in topological insulators with band engineering by chemical substitution and gate tuning.^[^ ^44^, ^45^ ^]^ With further tuning, the chemical potential will enter the FM gap (“Cut3” case) and a phase transition from the QHE to the QAHE is expected. A spectroscopic imaging technique, capable of accessing the wide energy range, such as STM may be helpful to study and trace the induced changes in the band structure.^[^ ^46^ ^]^

## 3 Conclusion

In conclusion, SdH quantum oscillations have been observed in pure and V‐doped Sb_2_Te_3_ thin films for both longitudinal and transverse magneto‐transport. The temperature‐dependent resistance and Landau fan diagrams demonstrate the strong band‐structure tailoring effect controlled by the V doping. This is similar to pure Sb_2_Te_3_, where angle‐dependent quantum oscillation studies confirmed the presence of topological surface states.^[^ ^24^, ^25^ ^]^ Furthermore, systematic transport studies for varying film thicknesses, doping concentrations, and bottom gate voltage provide insights on the band structure near the Fermi level. According to the Onsager relation, it is deduced that the still strong contributions from bulk bands across the Fermi level cause unquantized Hall resistance. However, V doping can be utilized for a controlled shift of the chemical potential, as indicated by the continuous reduction of the size of the 2D Fermi pockets. The work at hand may inspire further doping‐dependent studies, aiming at band engineering. By a depletion of the bulk carrier density, it will become possible to realize low‐field quantized Hall effect. Moreover, high mobility 2D surface states coexist with ferromagnetic order in the V‐doped samples. Therefore, more powerful band engineering, realized for example by chemical doping or electric gating methods, may be able to induce a crossover between the QHE and QAHE topological state. Hence, V‐doped Sb_2_Te_3_ provides a platform for the creation of novel quantum states. It can be used to build heterostructures to study exotic topological states, such as topological magnetoelectric effect, axion insulator and high‐Chern‐number insulator.^[^ ^47^, ^48^ ^]^ Nevertheless, the apparent distinct behavior of the doped samples in comparison to the pure ones suggests that further theoretical considerations are required for a full understanding.

## 4 Experimental Section

The experimental details are provided in the Supporting Information.

## Conflict of Interest

The authors declare no conflict of interest.

## Supporting information

Supporting Information

## Data Availability

The data that support the findings of this study are available from the corresponding author upon reasonable request.
