# Supplementary material for: Quantum Oscillations in Ferromagnetic (Sb, V)2Te3 Topological Insulator Thin Films
Source: Adv Mater. 2021 Aug 31;33(41):2102107. doi: 10.1002/adma.202102107 (PMC11469026; doi:10.1002/adma.202102107)
Supplement: Supplementary file 1 — Supporting Information [file ADMA-33-2102107-s001.pdf]

© 2021 Wiley-VCH GmbH

ADVANCED MATERIALS

Supporting Information

for *Adv. Mater.,* DOI: 10.1002/adma.202102107

Quantum Oscillations in Ferromagnetic (Sb, V)$_2$Te$_3$ Topological Insulator Thin Films

Liguo Zhang, Toni Helm,* Haicheng Lin, Fengren Fan, Congcong Le, Yan Sun, Anastasios Markou, and Claudia Felser**

Supplementary Information

Quantum Oscillations in Ferromagnetic (Sb, V)$_2$Te$_3$ Topological Insulator Thin Films

Liguo Zhang, Toni Helm*, Haicheng Lin, Fengren Fan, Congcong Le, Yan Sun, Anastasios Markou, Claudia Felser**

Part 1: Films preparation and characterization

The (Sb, V)$_2$Te$_3$ films are MBE-grown on the substrates by co-evaporation of Sb (99.999%), Te (99.999%), and V (99.5%) sources. The vacuum background of the MBE chamber was about 4×10^{-11} mbar. All the sources were evaporated with the Knudsen cells. During the growth, the flux ratio of the Te and (Sb, V) was about 20:1. The substrates are semi-insulating InP (111)B (Fe doped) and undoped STO(111). The substrate was kept at 240°C during film growth. A post-annealing process at the same temperature was performed after the growth to improve the film quality. The thickness of the film was determined by the ω-2θ diffractogram of the symmetric (0 0 6) peak in the XRD data of the film shown in **Figure S1**. The transport measurements were conducted in a 14 T. Quantum Design PPMS and a 65 T pulse-field magnet at the Dresden High Magnetic Field Laboratory. All the measurements use the standard 4-probe AC lock-in method.

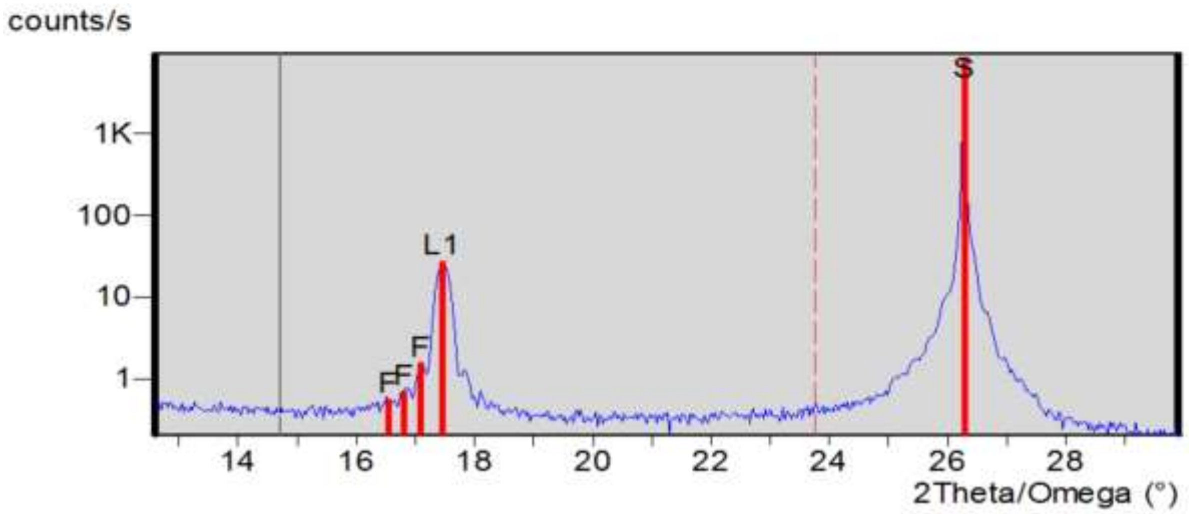

Figure S1 The ω-2θ diffractogram of the symmetric (0 0 6) peak of $Sb_{1.93}V_{0.07}Te_3$

Part 2: Temperature dependent resistances of varying samples

Figure S2 show the temperature-dependent zero-field resistance of different films. All the doped films exhibit the same overall shape distinct from the pure film. All the curves are normalized with by the reference values at 300 K. With increasing the doping concentration, the transition temperature shifts to higher temperature.

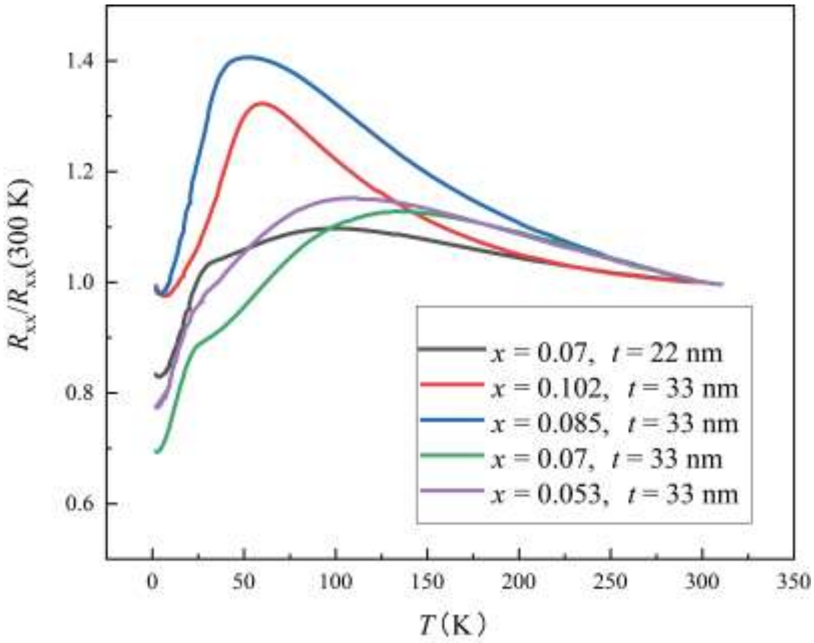

Figure S2 Temperature dependence of the zero-field resistance of doping series of $Sb_{2-x}V_xTe_3$ films with varying thickness *t*.

Part 3: Gaps extracted by fitting *RT* curves

A simple fitting method was used to estimate the gap with the formulism [1]:

$$R\ (T) = R_0 * e^{-\Delta/kT} \qquad (1)$$

The fitting results are shown in Figure S3. The extracted gaps are all in the level of only a few meV, and show no big change. This gap is possibly caused by the V-3*d* impurity.[2, 3]

The slope changes in *R*(*T*) indicate a complex evolution caused by the changes in the band structure and magnetic order. Further studies are needed for a better understanding.

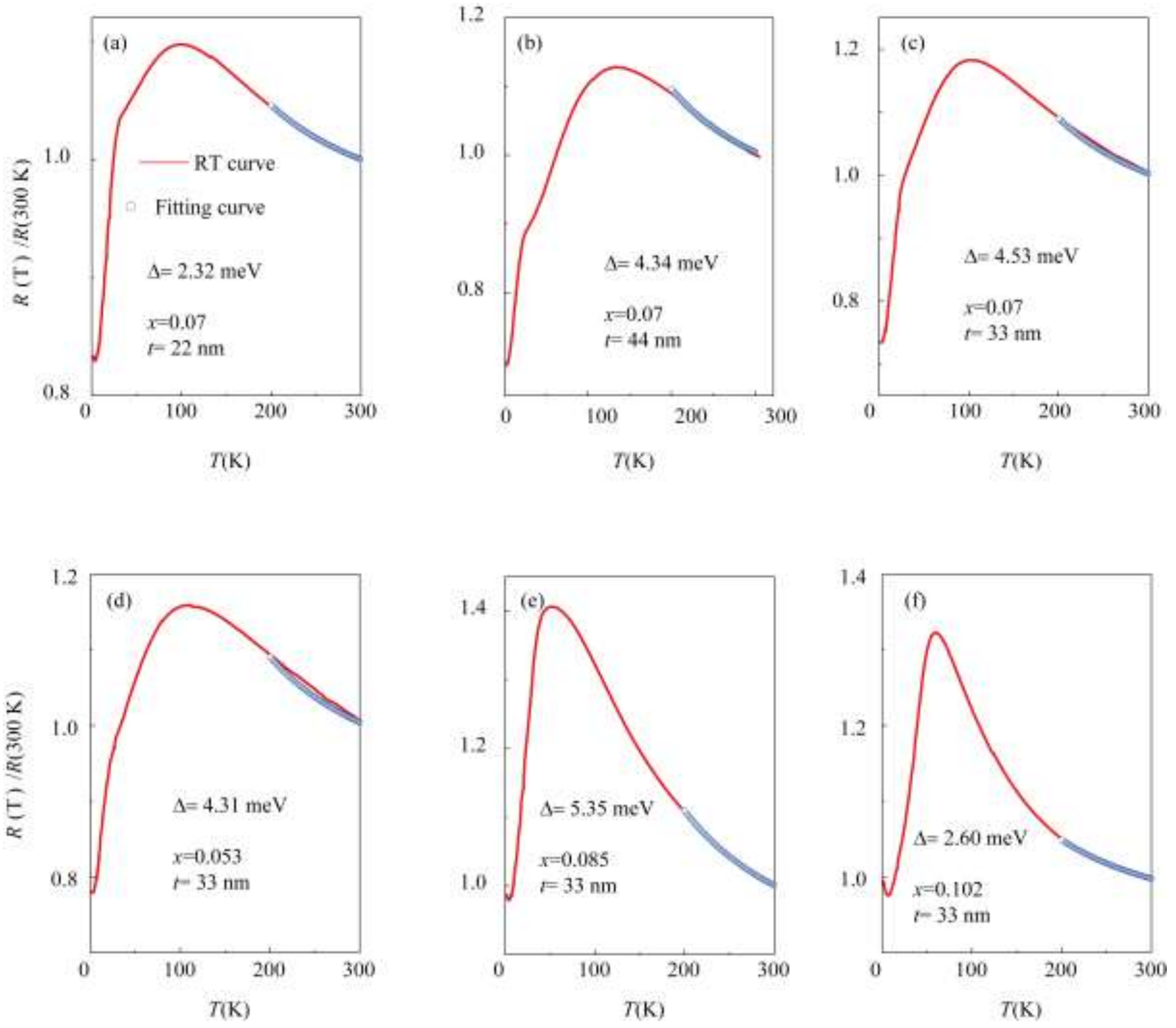

Figure S3 *RT* curve fitting according to Eq. 1

Part 4: Fitting method to extract the oscillation signals

As the quantum oscillation signals are superimposed to a huge slowly varying background a low-order polynomial is fitted and subtracted off the raw data. As the resistances of the doped films have hysteresis loop at low field, the fitting range is select above the saturated field. Adopting a similar approach,[4] **Figure S4** (a) illustrates the 3rd polynomial fitting on the doped sample in Figure 2b in main text. In order to elusive the feasibility of using 3rd polynomial fitting, in Figure S4 (b), the SdH oscillation signals got with 3rd and 4th polynomial is plotted respectively to contrast. With different orders, the results are close, so that it confirms that our method is appropriate. For the measurements in Figure 2h in main text, as the curve is more complex, the fittings are performed in two parts, see in Figure S4 (c) and (d). For the fitting of the Landau fan diagram, the positions of valleys in both undoped and doped samples are set to integer numbers and the peaks positions are the half integer numbers.[4,5] The reason is that, in both extracted SdH oscillation curves, the peaks and valleys potions of the longitudinal and transverse channels are almost located at the same points.

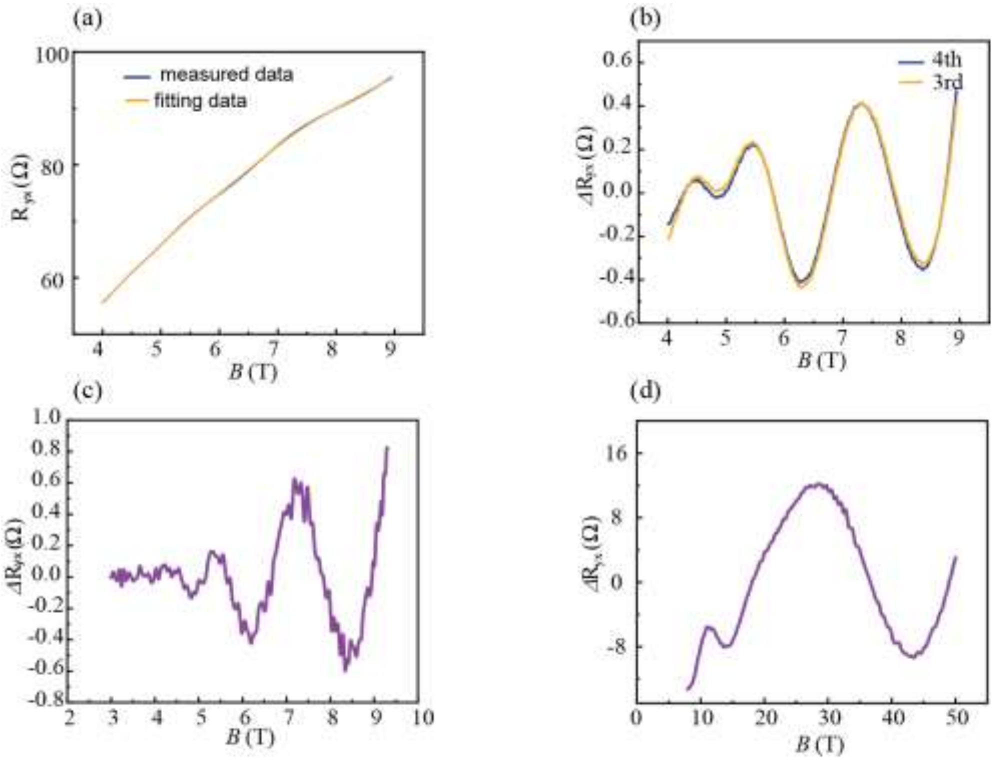

Figure S4 **(a)** 3rd order polynomial fit to the raw Hall data of the doped sample. **(b)** The SdH oscillation signals obtained after subtracting 3rd and 4th order polynomial fits. **(c)** The SdH oscillation signal obtained by subtracting 3rd order polynomial for fields below 9.5 T **(d)** The SdH oscillation signals obtained by subtracting 3rd order polynomial for fields between 8 and 50 T.

Part 5: Phase difference in longitudinal and Hall channels

In the main text, the phase of the oscillation signals (Figures 2e and 2f) between longitudinal and transverse resistance are all most the same, which is unusual. We indeed observe deviations between the phases of oscillations in the longitudinal resistance and Hall resistance. In Figure S5, we give two examples and different phase difference.

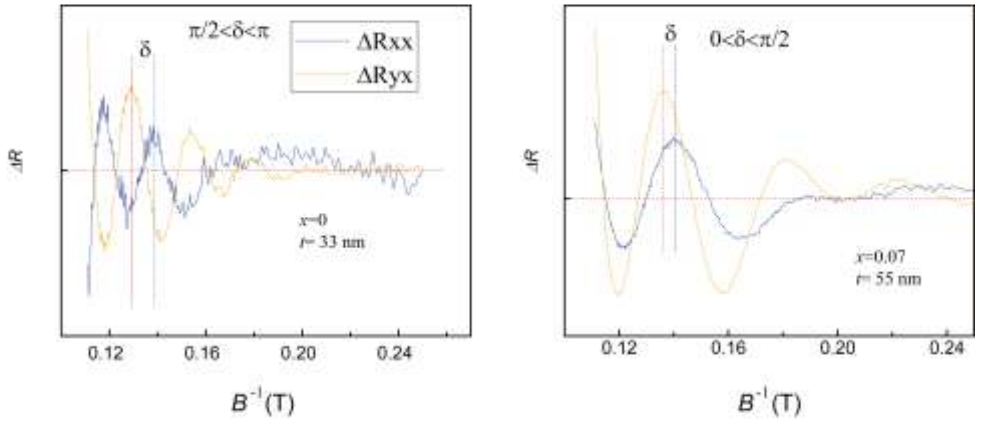

Figure S5 Two oscillation examples of the phase relationship between longitudinal and transverse resistance in undoped sample (left), and (right) for a doped sample.

Part 6: Gating effect _period summary

In this part, the evolution of a few quantities is plotted to understand the gating effect.

The effective carrier densities of pure Sb_2Te_3 and doped $Sb_{1.93}V_{0.07}Te_3$, mentioned in the main text in Figure 3, recorded for low magnetic fields are plotted in **Figure 6** (a) and (b). In both samples, the amplitude is largest for V_G around 200 V. However, the oscillation frequency hardly changes, see in Figure S6 (c) and (d). The main reason is that the density of states of bulk bands near the Fermi level is very large and only a small shift can be achieved by gate tuning.

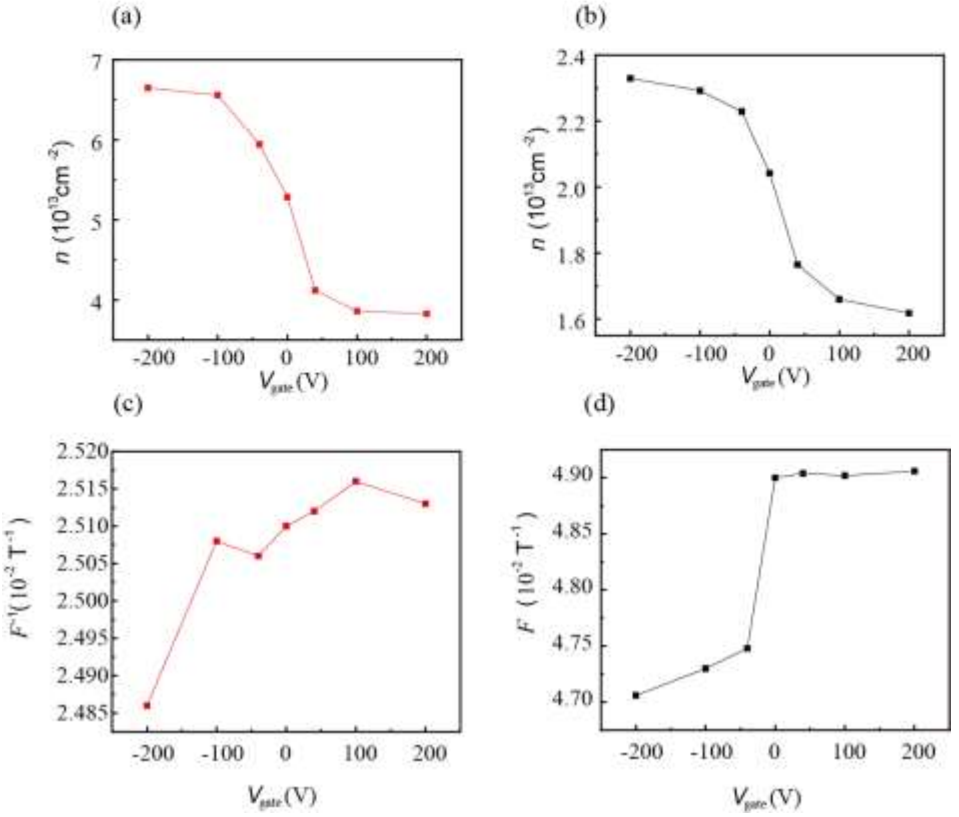

Figure S6 (a) and (b) The effective Hall carrier density and **(c) and (d)** the period of the quantum oscillations depending on V_G for the pure and x = 0.07 film with t = 33 nm**.**

Part 7: Hall data

All the doped samples in our studies show ferromagnetism with the typical anomalous Hall effect. Figure S7 shows the Hall data of doped samples with different V-doping levels (t =33 nm) with typical hysteresis loop.

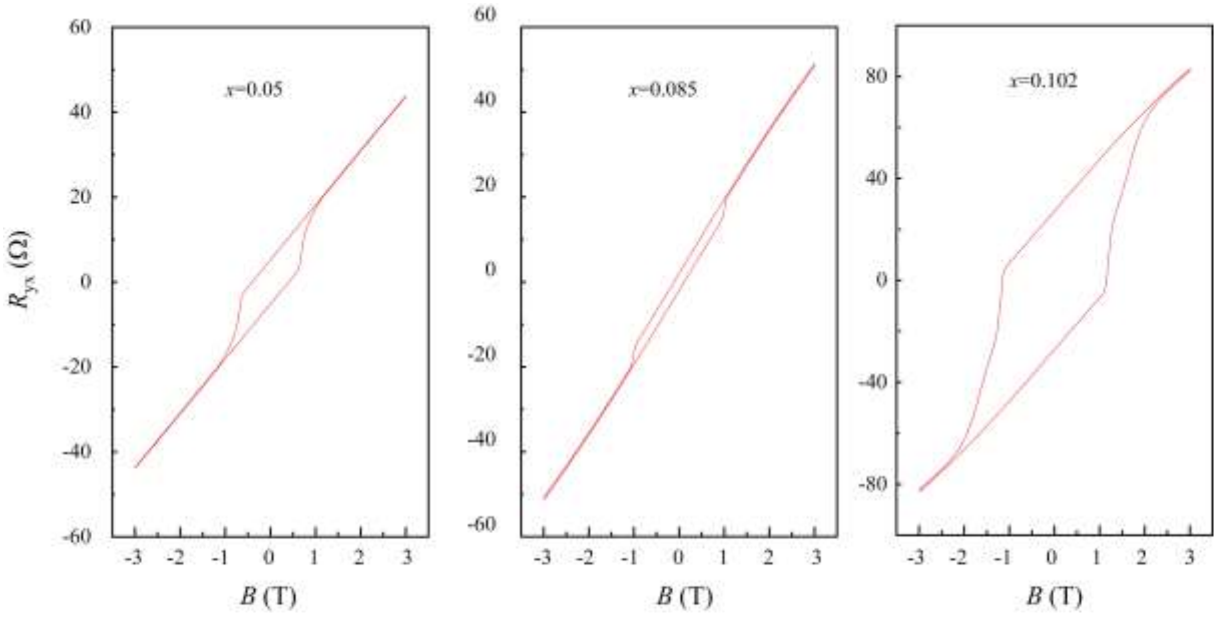

Figure S7 Hall data in doped samples (at 2 K)

Part 7: The list of samples and their properties

Table S1 Samples parameters and corresponding figures

Index	substrate	Thickness (nm)	Doping level x	Oscillation Frequency (T)	corresponding figures
1#	InP	44	0	36.5	Figure 2 (a)
2#	InP	44	0.07	23.2 /22	Figure 2 (b), (f)
3#	$SrTiO_3$	33	0	40.4	Figure 3(a), Figure 4(c)
4#	$SrTiO_3$	22	0.07	20.4	Figure 3(e)
5#	$SrTiO_3$	11	0.07	--	Figure 4(a)
6#	$SrTiO_3$	22	0.07	23.0	Figure 4(a), (e)
7#	$SrTiO_3$	33	0.07	23.2	Figure 4(a), (c)
8#	$SrTiO_3$	44	0.07	23.3	Figure 4(a)
9#	$SrTiO_3$	55	0.07	24.2	Figure 4(a)
10#	$SrTiO_3$	33	0.053	30.7	Figure 4(c)
11#	$SrTiO_3$	33	0.085	18.0	Figure 4(c)
12#	$SrTiO_3$	33	0.102	16.9	Figure 4(c)

References

[1] W. Zhao, L. Chen, Z. Yue, Z. Li, D. Cortie, M. Fuhrer, X. Wang, *npj Quantum Mater*. **2019**, *4*, 56.

[2] T. R. F. Peixoto, H. Bentmann, S. Schreyeck, M. Winnerlein, C. Seibel, H. Maaß, M. Al-Baidhani, K. Treiber, S. Schatz, S. Grauer, C. Gould, K. Brunner, A. Ernst, L. W. Molenkamp, F. Reinert, *Phys. Rev. B* **2016**, *94*, 195140.

[3] Z. Ren, A. A. Taskin, S. Sasaki, K. Segawa, Y. Ando, *Phys. Rev. B*. **2010**, *82*, 241306.

[4] C. Wyrich, T. Merzenich, J. Kampmeier, I. E. Batov, G. Mussler, J. Schubert, D. Grützmacher, Th. Schäpers, *Appl. Phys. Lett.* **2017**, *110*, 092104.
[5] A. Taskin and Y. Ando, *Phys. Rev. B* **2011**, *84*, 035301.
